# A Rolling Bearing Fault Diagnosis Method Based on Wild Horse Optimizer-Enhanced VMD and Improved GoogLeNet

**DOI:** 10.3390/s25144421

**Published:** 2025-07-16

**Authors:** Xiaoliang He, Feng Zhao, Nianyun Song, Zepeng Liu, Libing Cao

**Affiliations:** 1 School of Mechanical Engineering, Southeast University, Nanjing 211189, China; 230219115@seu.edu.cn; 2 College of Design and Engineering, National University of Singapore, Singapore 117576, Singapore; 3 School of Engineering, Newcastle University, Newcastle upon Tyne NE1 7RU, UK; f.zhao8@newcastle.ac.uk (F.Z.); zepeng.liu@newcastle.ac.uk (Z.L.); 4 School of Artificial Intelligence, Beijing Normal University, Beijing 100875, China; 202331081003@mail.bnu.edu.cn

**Keywords:** improved wild horse optimizer (IWHO), variational mode decomposition (VMD), improved GoogLeNet, fault diagnosis

## Abstract

To address the challenges of weak fault features and strong non-stationarity in early-stage vibration signals, this study proposes a novel fault diagnosis method combining enhanced variational mode decomposition (VMD) with a structurally improved GoogLeNet. Specifically, an improved wild horse optimizer (IWHO) with tent chaotic mapping is employed to automatically optimize critical VMD parameters, including the number of modes *K* and the penalty factor *α*, enabling precise decomposition of non-stationary signals to extract weak fault features. The vibration signal is decomposed, and the top five intrinsic mode functions (IMFs) are selected based on the kurtosis criterion. Time–frequency features are then extracted from these IMFs and input into a modified GoogLeNet classifier. The GoogLeNet structure is improved by replacing standard *n* × *n* convolution kernels with cascaded 1 × *n* and *n* × 1 kernels, and by substituting the ReLU activation function with a parameterized TReLU function to enhance adaptability and convergence. Experimental results on two public rolling bearing datasets demonstrate that the proposed method effectively handles non-stationary signals, achieving 99.17% accuracy across four fault types and maintaining over 95.80% accuracy under noisy conditions.

## 1. Introduction

Under the combined influence of multiple factors, including internal system conditions and external environments, the performance of electromechanical products gradually degrades, eventually leading to failures [1,2,3]. Rolling bearings are rotating components that play a crucial role in transmitting motion and power in electromechanical products. Conducting condition monitoring and fault diagnosis for rolling bearings is essential to ensure the normal operation of electromechanical products and reduce or prevent accidents [4]. It is well known that vibration signals can effectively reflect the operating state of rotating machinery and serve as an important source of information for fault diagnosis [5].

Generally, fault diagnosis consists of three stages: signal acquisition, signal processing, and fault identification [6]. Among these, time–frequency analysis is suitable for processing non-stationary signals and can reveal the variation patterns of vibration characteristics over time. However, traditional signal processing methods still have limitations. For example, Fourier transform cannot effectively correlate time-domain and frequency-domain signals, while wavelet transform, although capable of extracting weak features, suffers from poor adaptability [7]. Therefore, selecting or designing appropriate algorithms is crucial for better extraction of fault-related feature signals.

Empirical mode decomposition (EMD) is suitable for analyzing non-stationary signals without requiring predefined basis functions [8]. Liang et al. [9] applied the EMD method to centrifugal pump fault diagnosis, considering the impact of strong noise environments on EMD. However, the EMD method suffers from mode mixing and end effects, and some intrinsic mode functions (IMFs) may contain multiple time-scale components, leading to the ineffective extraction of fault features. To mitigate the mode mixing phenomenon, Zhao et al. [10] introduced the ensemble empirical mode decomposition (EEMD) method, but it also causes amplitude variations and introduces reconstruction errors. Reference [11] proposed the variational mode decomposition (VMD) method, which overcomes the limitations of EMD, adaptively decomposes signals, and effectively extracts vibration signal features, making it particularly suitable for processing nonlinear and non-stationary signals.

Research has shown that the input parameters significantly influence the decomposition performance of VMD [12]. The penalty factor *α* affects the bandwidth of the modes. An excessively small *α* may lead to mode mixing, while a large *α* helps avoid mixing but may cause the loss of local information. The mode number *K* directly determines the number of decomposed modes. An overly large *K* may generate spurious modes, whereas an insufficient *K* can result in under-decomposition, failing to fully extract hidden features. Most existing studies determine the optimal values of α and *K* through repeated experiments, but such methods primarily rely on subjective judgment and lack objective evaluation criteria.

To achieve adaptive parameter optimization, intelligent algorithms have been introduced into the fields of signal processing and fault diagnosis. Hua et al. [13] employed an improved artificial fish swarm algorithm to optimize VMD parameters for wind turbine fault diagnosis. Qi et al. [14] utilized the grey wolf optimizer to tune VMD parameters, addressing the issue of blind parameter selection. Xiong et al. [15] incorporated the sparrow search algorithm into long short-term memory networks to realize automatic hyperparameter optimization. In 2021, Naruei proposed a novel swarm intelligence optimization algorithm—the wild horse optimizer (WHO) [16]. Currently, the wild horse algorithm has been studied and applied in various domains, including parameter identification and fault diagnosis [17,18].

Feature extraction and fault pattern recognition constitute the core components of fault diagnosis. Commonly used methods include K-nearest neighbor (KNN), support vector machine (GoogLeNet), and artificial neural network (ANN) approaches [19,20]. The KNN method demonstrates high classification accuracy and insensitivity to outliers, yet suffers from computational complexity and inability to interpret the intrinsic meaning of data. ANNs possess strong self-learning and generalization capabilities with good adaptability, but are challenged by numerous parameters, optimization difficulties, and lengthy training times. GoogLeNet has gained attention in fault diagnosis as it effectively overcomes ANN’s limitations of slow learning speed and overfitting problems.

This paper proposes a novel fault diagnosis method that integrates an improved wild horse optimizer (IWHO) with an improved GoogLeNet network to address the challenges in vibration signal analysis for rolling bearing fault detection. The IWHO algorithm improves the original wild horse optimizer by incorporating tent chaotic mapping during population initialization, which increases diversity and avoids premature convergence. It is employed to adaptively determine the optimal values for key VMD parameters (namely, the number of decomposition modes *K* and the penalty factor *α*) by minimizing envelope entropy. The optimized VMD then decomposes vibration signals into IMFs, from which the top components are selected based on the kurtosis criterion. Time–frequency domain features are extracted from these IMFs to construct discriminative feature vectors. These features are subsequently input into an improved GoogLeNet model, where the standard *n* × *n* convolutions are replaced with more efficient 1 × *n* and *n* × 1 kernel pairs to reduce complexity, and the ReLU activation function is replaced by a trainable TReLU activation to enhance adaptability and convergence. This integrated approach ultimately enables accurate and robust fault classification.

The rest of the paper is organized as follows. Section 2 presents the framework of the proposed IWHO-VMD-GoogLeNet method and describes each part in detail. Section 3 illustrates the effectiveness and superiority of the proposed method in rolling bearing fault datasets. Conclusions and future directions are provided in Section 4.

## 2. Algorithm

### 2.1. IWHO-VMD

#### 2.1.1. VMD

**Figure 1:**
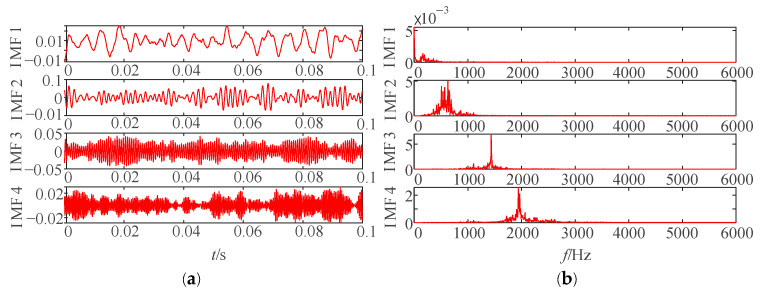
Time-domain waveforms and frequency spectra after VMD. (**a**) Time-domain waveforms. (**b**) Frequency spectra.

In 2014, Dragomiretskiy et al. proposed the VMD method [11]. VMD is an adaptive decomposition approach particularly suitable for processing nonlinear and non-stationary signals. Figure 1 shows the time-domain waveforms and frequency spectra of the first four IMFs of a signal after VMD.

The VMD method presets the decomposition number *K*, then determines the center frequencies and bandwidths of each modal component by seeking optimal solutions, ultimately decomposing the original signal *x*(*t*) into *K* modal components *u_k_*(*t*), where *k* = 1, 2, ⋯, *K*. The fundamental steps are as follows:

(1) Perform Hilbert transform on the modal components to obtain the unilateral spectrum:

(1) $$S_{k}=\left(\delta (t)+\frac{j}{\pi t}\right)u_{k}(t)$$

where *δ*(*t*) is the Dirac delta function.

(2) Shift the spectrum of each modal component *u_k_* (*t*) to its corresponding baseband:

(2) $$S_{fk}=\left[\left(\delta (t)+\frac{j}{\pi t}\right)u_{k}(t)\right]e^{-j\omega_{k}t}$$

where *ω_k_* represents the center frequency of *u_k_* (*t*).

(3) Estimate the bandwidth through Gaussian smoothing of the demodulated signal and construct the constrained variational model:

(3) $$\left\{\begin{array}{l} \underset{\left\{u_{k}(t)\right\}}{\min}\left\{\left\{w_{\left.w_{k}\right\}}\left\{\overset{K}{\underset{k=1}{\sum {\left\|\partial_{t}\left[\left(\delta (t)+\frac{j}{\pi t}\right)u_{k}(t)\right]e^{-j\omega_{k}t}\right\|}_{2}^{2}}}\right\}\right.\right.\\ \;\mathrm{s.t.}\;\overset{K}{\underset{k=1}{\sum }}u_{k}(t)=x(t) \end{array}\right.$$

where *∂_t_* denotes the partial derivative with respect to *t*, and ${\left\|\;\right\|}_{2}^{2}$ represents the *L*^2^ norm.

To solve this variational problem, penalty factor *α* and Lagrangian multiplier *λ* are introduced to transform the constrained problem into an unconstrained one, yielding the augmented Lagrangian expression:

(4) $$\begin{array}{c} L\left(\left\{u_{k}(t)\right\},\left\{\omega_{k}\right\},\lambda (t)\right)=\\ \;\alpha \sum_{k=1}^{K}{\left\|{𝜕}_{t}\left[\left(\delta (t)+\frac{j}{\pi t}\right)u_{k}(t)\right]e^{-j\omega_{k^{t}}t}\right\|}_{2}^{2}+\\ \;{\left\|x(t)-\sum_{k=1}^{K}u_{k}(t)\right\|}_{2}^{2}+〈\lambda (t),x(t)-\sum_{k=1}^{K}u_{k}(t)〉 \end{array}$$

The solution is obtained through alternating direction method of multipliers (ADMM) by iteratively updating $u_{k}^{n+1}$, $\omega_{k}^{n+1}$, and $\lambda_{k}^{n+1}$ until meeting the convergence criterion:

(5) $$\sum_{k}\left({\left\|u_{k}^{n+1}-u_{k}^{n}\right\|}_{2}^{2}/{\left\|u_{k}^{n}\right\|}_{2}^{2}\right)<\varepsilon$$

where *ε* indicates the convergence tolerance.

The update equations for individual variables during iteration are:

(6) $${\hat{u}}_{k}^{n+1}(\omega )=\frac{\hat{x}(\omega )-\sum_{i=1}^{k-1}{\hat{u}}_{i}^{n+1}(\omega )-\sum_{i=k+1}^{K}{\hat{u}}_{i}^{n}(\omega )+\frac{{\hat{\lambda }}^{n}(\omega )}{2}}{1+2\alpha {\left(\omega -\omega_{k}^{n}\right)}^{2}}$$

(7) $$\omega_{k}^{n+1}=\frac{\int_{0}^{\infty }\omega {\left|{\hat{u}}_{k}^{n+1}(\omega )\right|}^{2}d\omega }{\int_{0}^{\infty }{\left|{\hat{u}}_{k}^{n+1}(\omega )\right|}^{2}d\omega }$$

(8) $${\hat{\lambda }}^{n+1}(\omega )={\hat{\lambda }}^{n}(\omega )+\tau \left(\hat{x}(\omega )-\sum_{k=1}^{K}{\hat{u}}^{n+1}(\omega )\right)$$

where *n* is a positive integer; ^ denotes the Fourier transform; and *τ* represents the fidelity coefficient.

#### 2.1.2. IWHO

**Figure 2:**
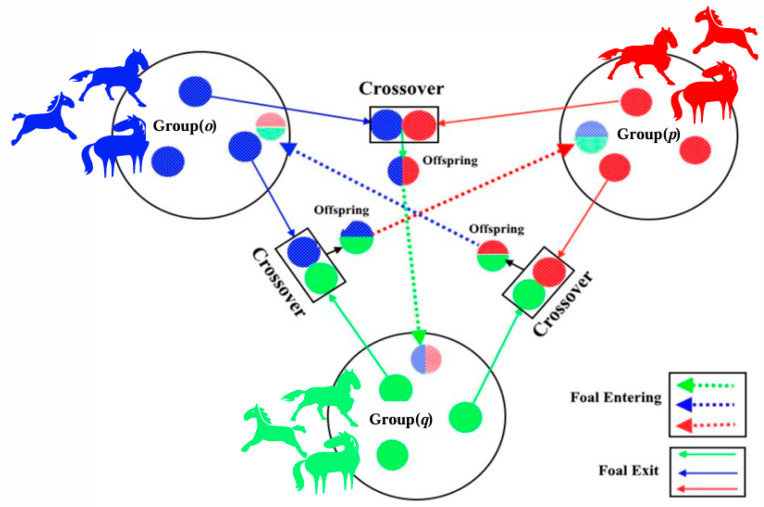
The schematic diagram of WHO.

In 2021, Iraj Naruei and Farshid Keynia proposed the WHO inspired by the social behaviors of wild horse populations [16]. The schematic diagram of WHO is shown in Figure 2.

As shown in Figure 2, a horse herd consists of stallions (dominant males), mares, and foals, where stallions act as leaders while others follow. Herd behaviors include grazing, leadership hierarchy, and mating activities. Stallions maintain proximity to mares, while foals gradually become independent as they mature. The WHO algorithm optimizes objectives by simulating these herd dynamics. For detailed mathematical formulations of WHO’s grazing, leadership, mating, and role-switching behaviors, refer to [16].

The original WHO initializes populations randomly within search boundaries, which may lead to homogeneity and suboptimal performance. This study enhances WHO by incorporating tent chaotic mapping during population initialization [17]. Chaotic mapping, which is a dynamic system exhibiting uncertainty and complexity, generates sequences that avoid local clustering issues inherent in conventional random initialization. Integrating tent chaotic mapping into WHO provides the following: (1) broader exploration of the search space during initialization; (2) guaranteed population diversity; (3) prevention of premature convergence. The tent chaotic sequence *E_I_*_, *G*_ is generated as:

(9) $$E_{I+1,G}=\left\{\begin{array}{cc} E_{I,G} & 0\leq E_{I,G}\leq u\\ \frac{1-E_{I,G}}{1-u} & u\leq E_{I,G}\leq 1 \end{array}\right.$$

where *I* represent the current iteration number, *G* is the number of wild horse groups, and *u* is a random number between [0, 1].

**Figure 3:**
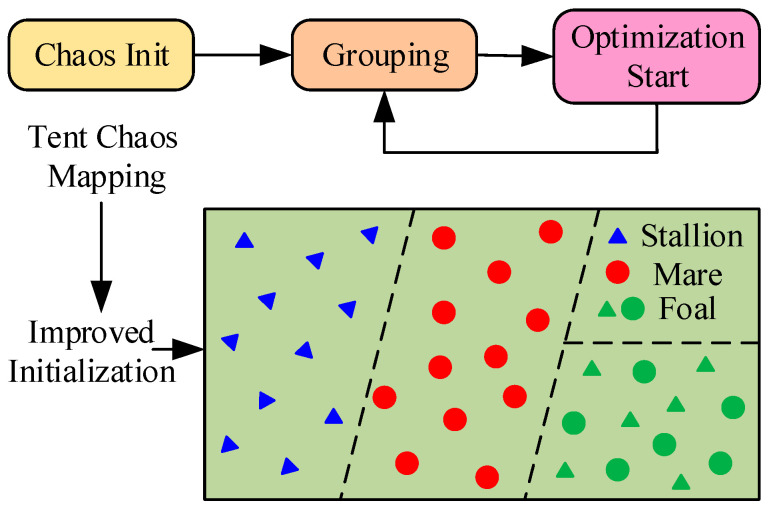
IWHO initialization with tent chaotic mapping.

As seen in Figure 3, based on *E_I_*_, *G*_, the initial sequence of wild horse population individuals within the search range is generated as:

(10) $$H_{I,G}=H_{I,G}^{\max}+E_{I,G}(H_{I,G}^{\max}-H_{I,G}^{\min})$$

where $H_{I,G}^{\max}$ and $H_{I,G}^{\min}$ represent the maximum and minimum values of the column, respectively. The initial population is divided into several groups, with *N* individuals in total. The number of groups led by stallions is *S*, and the proportion of stallions is defined as *PS* (i.e., *S*/*N*). The number of followers (mares and foals) equals *N*-*S*.

#### 2.1.3. VMD Parameter Auto-Tuning Model Based on IWHO

The decomposition performance of VMD is significantly influenced by its input parameters [12,13]. This study employs IWHO to automatically optimize VMD parameters (modal number *K* and penalty factor *α*), using minimum envelope entropy as the fitness function. Envelope entropy (*H_e_*), an effective metric for non-stationary signal analysis, quantifies signal complexity. The *H_e_* is defined as:

(11) $$H_{e}=-\sum_{i=1}^{k}\left(\frac{\left|e_{i}(t)\right|}{\sum_{i=1}^{k}\left|e_{i}(t)\right|}\right)\ln\left(\frac{\left|e_{i}(t)\right|}{\sum_{i=1}^{k}\left|e_{i}(t)\right|}\right)$$

where $e_{i}(t)$ represents the envelope signal of the *i*-th mode.

The automatic parameter optimization of VMD aims to determine the optimal mode number *K* and penalty factor *α* by minimizing the envelope entropy:

(12) $$\begin{array}{l} \underset{k,\alpha }{\min}H_{e}(K,\alpha )\\ \mathrm{s.t.}\;\;\begin{array}{l} K\in [1,15]\\ \alpha \in [500,4000] \end{array} \end{array}$$

### 2.2. Kurtosis Criterion and Time–Frequency Feature Extraction

The vibration signal *x*(*t*) is decomposed via IWHO-VMD to obtain multiple IMFs arranged from high to low frequencies. Extracting fault-sensitive feature vectors from these IMFs constitutes a critical step in signal processing. Research demonstrates that higher kurtosis values indicate stronger impulse components in the signal, corresponding to increased probability of bearing faults [21]. This study therefore adopts the kurtosis criterion for IMF selection.

As a dimensionless parameter sensitive to impulse signals, kurtosis is particularly suitable for diagnosing surface-damage faults. Its mathematical expression is given by [17]

(13) $$Q=\frac{E{\left(x-\mu \right)}^{4}}{\sigma^{4}}$$

where *x* represents the bearing fault signal, *μ* denotes the mean value, and *σ* is the standard deviation.

To effectively monitor equipment operational status in real-time, feature vector extraction is essential. Time-domain statistical features are widely employed in condition monitoring due to their computational efficiency and sensitivity to state variations. Common time-domain statistical features include mean value, standard deviation, and kurtosis. For a given signal *x* = [*x*_1_, *x*_2_, …, *x_m_*], the time—frequency domain features constituting the feature vector are summarized in Table 1 [22].

### 2.3. Improved GoogLeNet Classifier

**Figure 4:**
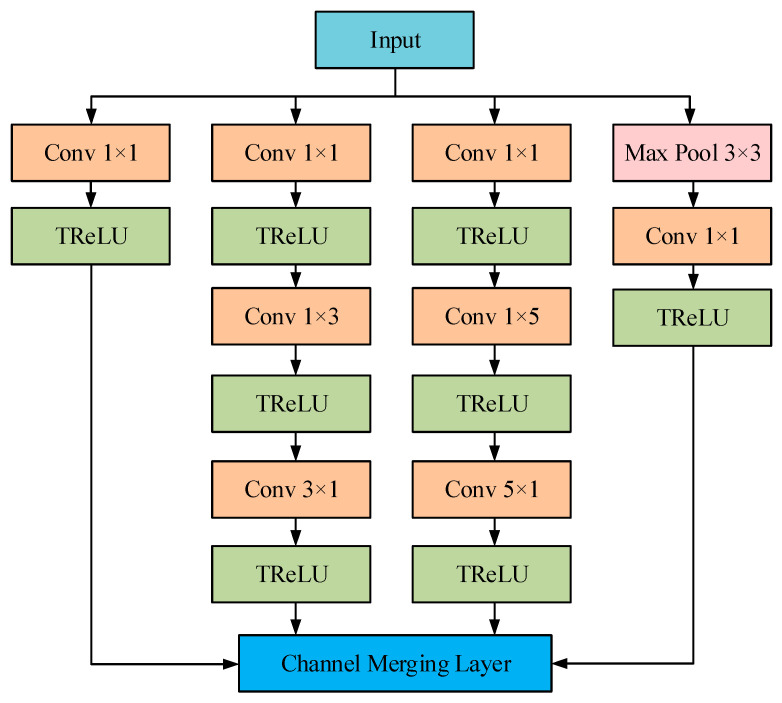
The structure of improved GoogLeNet.

Unlike traditional deep learning models that rely on increasing depth to enhance performance, GoogLeNet introduced Inception blocks to expand the model’s width [19]. This approach reduces the number of parameters while simultaneously improving recognition accuracy. Its original structure consists of 3 convolutional layers, 9 Inception blocks (a total of 18 convolutional layers), and 2 auxiliary classifiers. The structure is relatively large, with further details available in [23]. In this paper, some improvements to GoogLeNet were employed as follows (as shown in Figure 4):

(1) Replace the *n* × *n* size convolution kernel in the Inception block with a convolution combination of 1 × *n* and *n* × 1 to reduce the model parameters to adapt to small-scale datasets, while reducing the training time.
(2) The ReLU activation function is replaced by TReLU. By introducing trainable parameters *β* and *γ*, the adaptability of the activation function to different data distributions is improved and the convergence speed is accelerated. The formula for the TReLU activation function is

(14) $$\begin{array}{c} \mathrm{TReLU}(x)=e^{\beta }\mathrm{ReLU}(x)+\gamma \\ \mathrm{ReLU}(x)=\max(0,x) \end{array}$$

where *x* represents the input value through stratification; *β*, *γ* indicate trainable parameters; *e* is a natural constant of about 2.72.

### 2.4. Proposed Fault Diagnosis Framework

**Figure 5:**
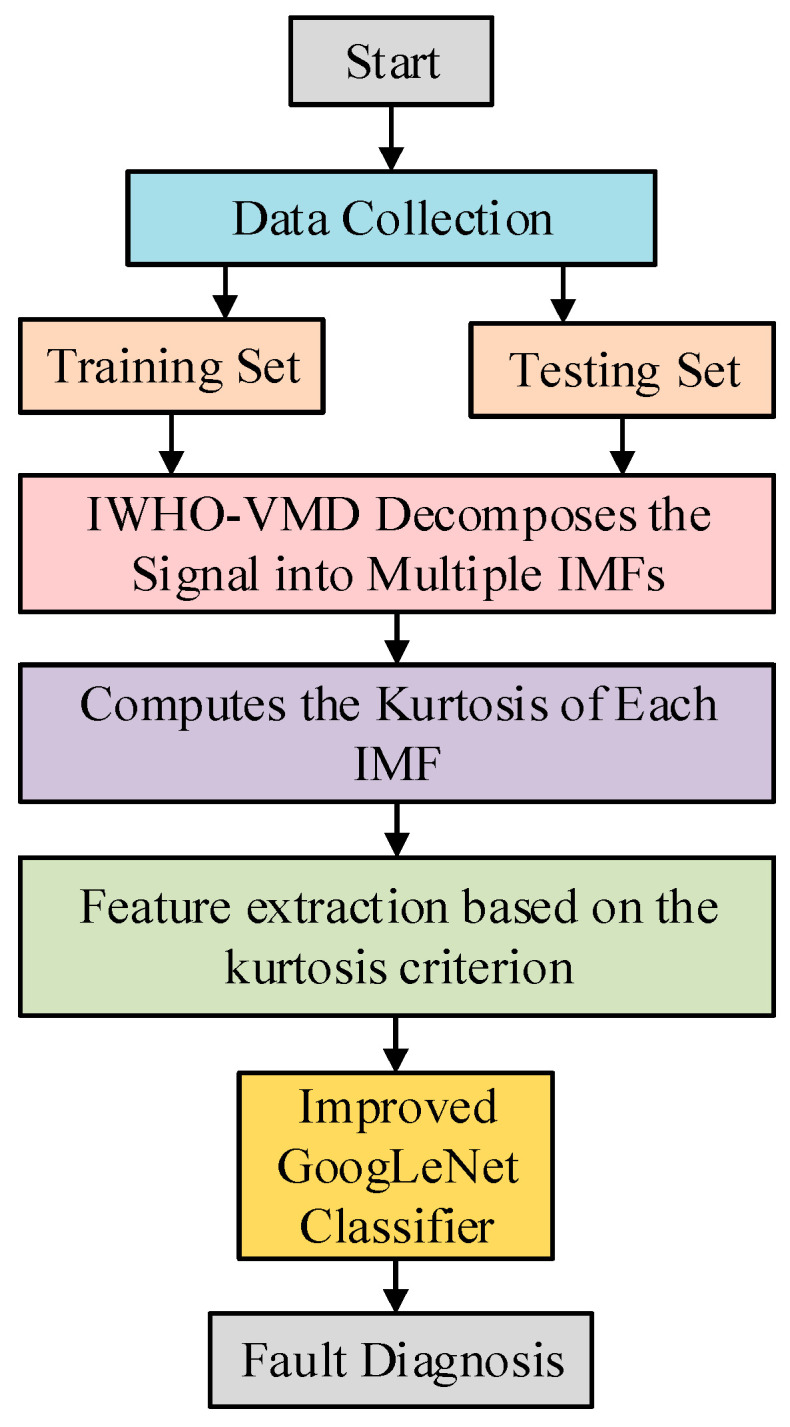
The workflow of the proposed IWHO-VMD-GoogLeNet fault diagnosis method.

This study proposes a fault diagnosis method integrating IWHO-VMD and improved GoogLeNet, with the following workflow (as shown in Figure 5):

(1) Vibration signals are collected under different machine states using sensors to reflect operational conditions.
(2) The acquired signals are decomposed via IWHO-VMD to obtain *Y* IMF components, with kurtosis values computed for each IMF.
(3) The top *y* IMFs with highest kurtosis values are selected based on the kurtosis criterion, followed by time–frequency feature extraction to capture fault characteristics.
(4) Feature vectors are constructed to train an GoogLeNet classifier for fault pattern differentiation. Among them, the dataset of each state is divided into a training set and a test set at a classical ratio of 7:3.
(5) Test samples are input into the trained GoogLeNet classifier for pattern recognition and final classification.

## 3. Case Study I: CWRU Dataset

### 3.1. Data Acquisition for Case Study I

**Figure 6:**
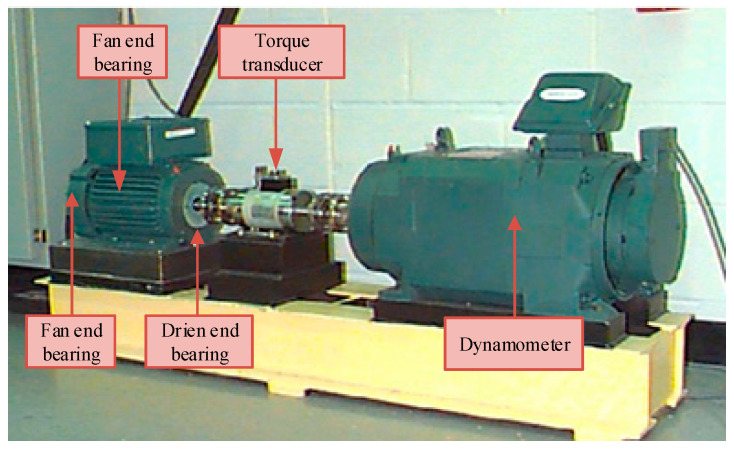
CWRU testing rig.

To validate the effectiveness of the proposed method, a case study was conducted based on fault test data of rolling bearings under the case western reserve university (CWRU). The testing rig is shown in Figure 6 [24]. The experimental platform consists of a 2-horsepower motor, a torque sensor/encoder, a power meter, and an electronic controller. The test bearings SKF6205 (SKF Group, Gothenburg, Sweden) were driven by a three-phase induction motor and instrumented with torque sensors and accelerometers. Detailly, the electric discharge machining technology is adopted to set single-point faults with diameters of 0.007, 0.014, and 0.021 inches on the inner race, outer race surface, and rolling elements of the bearings, respectively.

**Figure 7:**
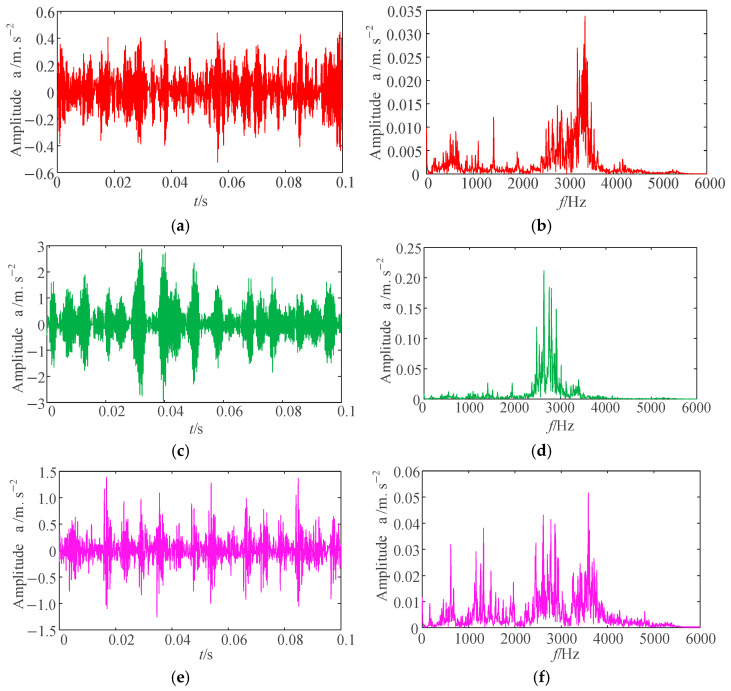

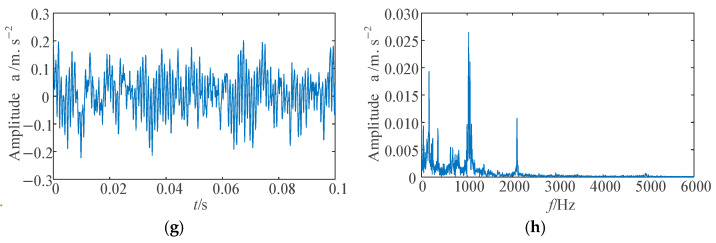
Time-domain waveforms and frequency spectra of rolling bearing under four operating conditions. (**a**,**b**) Rolling element fault. (**c**,**d**) Outer race fault. (**e**,**f**) Inner race fault. (**g**,**h**) Normal state.

In this testing rig, vibration data were acquired at a 12 kHz sampling rate under four operating conditions: normal state, inner race fault, outer race fault, and rolling element fault. For each condition, 100 vibration signal datasets were collected. The time-domain waveform of the raw signals from the first dataset is presented in Figure 7.

As shown in Figure 7, the time-domain waveforms of vibration signals under fault conditions exhibit significant differences from those under normal state. Furthermore, distinct impulse components are observed in the bearing signals for inner race faults, outer race faults, and rolling element faults. The frequency spectra reveal similar frequency components near 3000 Hz across different fault types. However, fault classification cannot be directly determined solely based on time-domain waveforms and frequency spectra.

### 3.2. IWHO Iteration Curves

**Figure 8:**
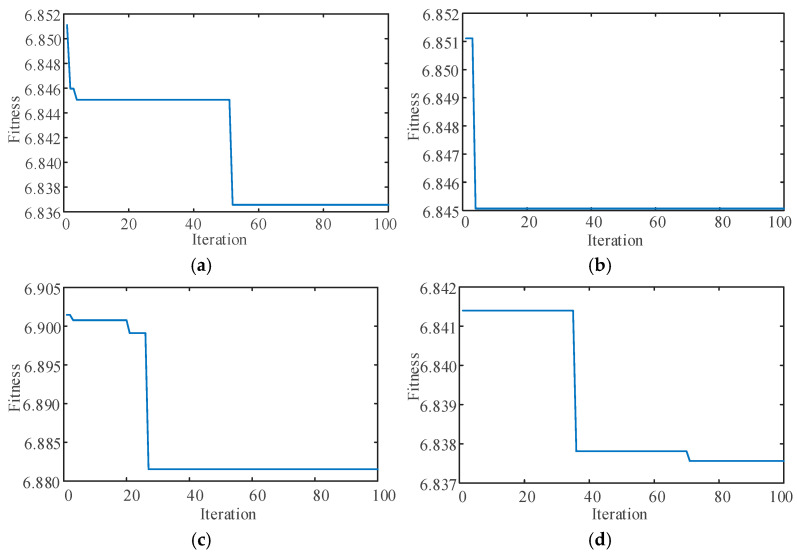
Iteration curves of WHO-optimized VMD parameters under four conditions. (**a**) Rolling element fault. (**b**) Outer race fault. (**c**) Inner race fault. (**d**) Normal state.

The IWHO was employed to automatically optimize VMD parameters, obtaining optimal *α* and *K* values for all four conditions. The IWHO iteration curves and optimization results for the four conditions are presented in Figure 8 and Table 2, respectively. The algorithm parameters were set as follows: population size = 20 and maximum iterations = 100.

As shown in Figure 7, the IWHO algorithm achieved convergence and obtained optimal solutions before reaching the maximum iteration count (100) for all four conditions. Notably, convergence was attained before the 40th iteration for the first three conditions. These results demonstrate the superior performance of IWHO in optimizing VMD parameters.

### 3.3. IMFs After Enhanced-VMD

**Figure 9:**
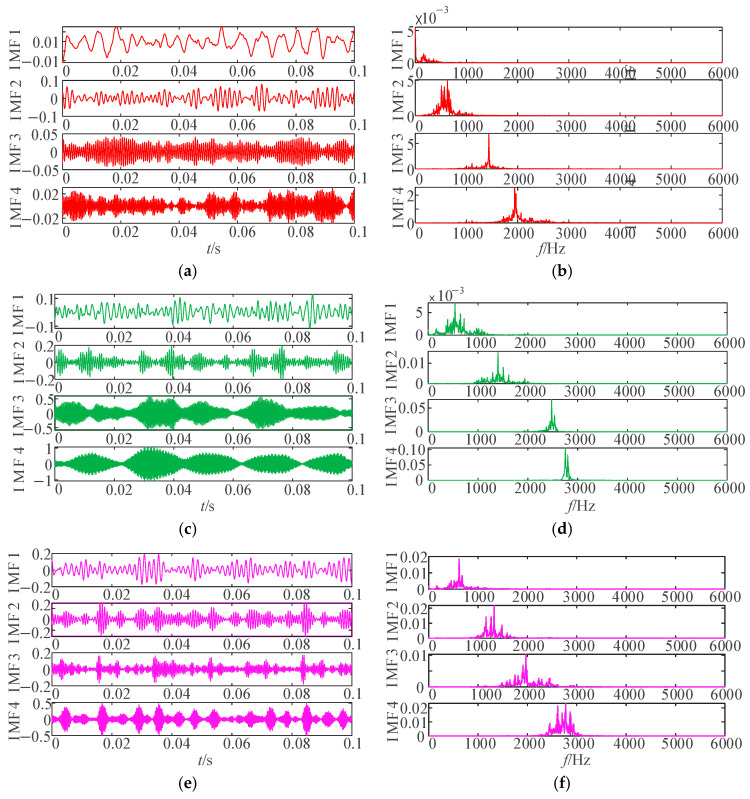

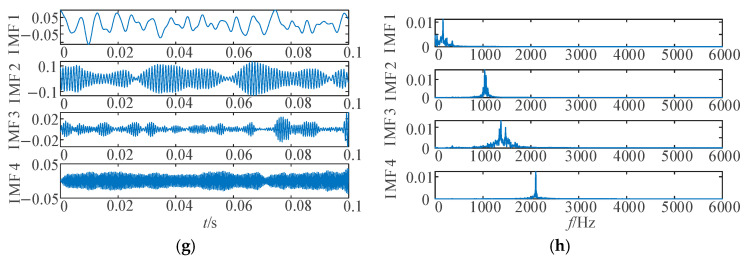
IMFs and their frequency spectra obtained through IWHO-VMD under four conditions. (**a**,**b**) Rolling element fault. (**c**,**d**) Outer race fault. (**e**,**f**) Inner race fault, (**g**,**h**) Normal state.

After parameter optimization via IWHO, the optimal parameters listed in Table 2 were applied to VMD, yielding the corresponding IMFs for each condition (as shown in Figure 9). For conciseness, only four IMFs are displayed.

As evidenced in Figure 9, the IWHO-VMD successfully separated vibration signals under different conditions into distinct numbers of IMFs. Both time-domain waveforms and frequency spectra demonstrate significant variations in frequency composition and distribution among the IMFs, confirming the superior decomposition performance achieved through IWHO-optimized VMD parameters.

### 3.4. Kurtosis Criterion Analysis

**Figure 10:**
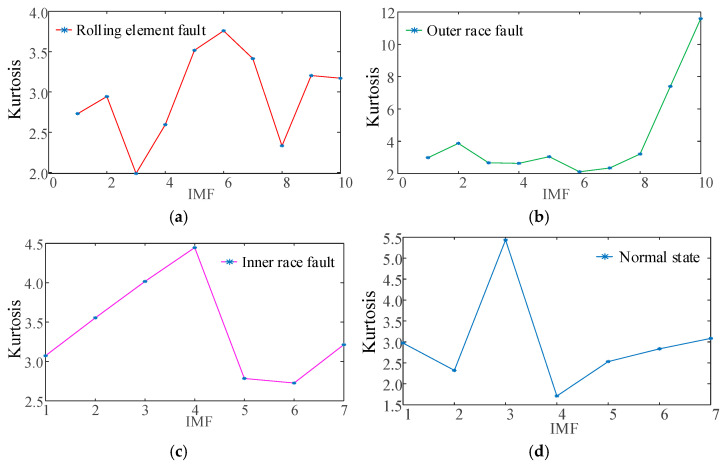
Kurtosis values of decomposed IMFs under four operating conditions. (**a**) Rolling element fault. (**b**) Outer race fault. (**c**) Inner race fault. (**d**) Normal state.

Figure 10 presents the kurtosis values calculated for the IMFs obtained through IWHO-VMD under each operational condition.

As illustrated in Figure 10, the kurtosis values of the IMFs vary significantly across different operational states. In accordance with the kurtosis criterion and repeated experimental validation, the five IMFs exhibiting the highest kurtosis values were selected for subsequent feature extraction. These IMFs were used to compute time-domain, frequency-domain, and time–frequency-domain features, which collectively constitute the feature vectors for fault diagnosis. The IMFs were first reordered in ascending sequence based on their kurtosis values, after which the top five IMFs with the highest kurtosis were identified as the effective decomposition modes for time-domain and frequency-domain feature extraction.

### 3.5. Fault Diagnosis Results

**Figure 11:**
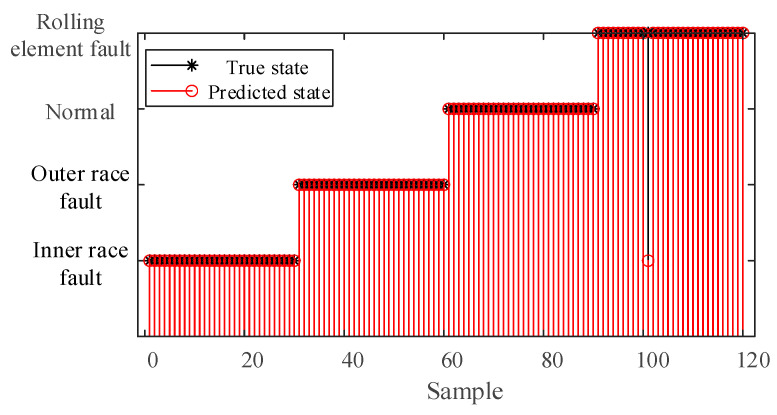
Fault diagnosis results of IWHO-VMD-GoogLeNet for case study I.

The constructed feature vectors were input into the GoogLeNet model for training to achieve fault diagnosis of rolling bearings. Among them, the dataset of each state is divided into a training set and a test set at a ratio of 7:3. The fault diagnosis results and their corresponding confusion matrix are presented in Figure 11.

The fault diagnosis results presented in Figure 11 demonstrate high classification performance across different conditions. All test samples corresponding to the normal state and inner race fault were correctly classified, achieving perfect diagnostic accuracy (100%). Similarly, the outer race fault condition exhibited 100% diagnostic accuracy. For the rolling element fault condition, 29 out of 30 test samples were accurately identified, with one case misclassified as inner race fault, yielding a diagnostic accuracy of 96.67%. Overall, the system achieved 99.17% diagnostic accuracy, with only one misclassification occurring among the total 120 test samples representing these four operational conditions.

### 3.6. Performance Comparison of Methods

To evaluate the effectiveness of the proposed IWHO-VMD-GoogLeNet method, a comparative analysis was conducted with three well-established fault diagnosis approaches: VMD-GoogLeNet, PSO-VMD-GoogLeNet, and WOA-VMD-GoogLeNet. The comparative results are summarized in Table 3.

The comparative results in Table 3 reveal three key findings about the diagnostic performance of different methods. First, the baseline VMD-GoogLeNet approach yields the lowest accuracy of 94.17%, while intelligent optimization algorithms (PSO, WOA, and IWHO) all demonstrate significant improvements in diagnostic performance. Second, the proposed IWHO algorithm exhibits marginally better optimization capability than both PSO and WOA, as evidenced by its higher accuracy gains. Most notably, the IWHO-VMD-GoogLeNet method achieves the highest fault diagnosis accuracy of 99.17%, representing a 5.31% improvement over VMD-GoogLeNet, a 3.49% enhancement compared to PSO-VMD-GoogLeNet, and a 2.59% increase versus WOA-VMD-GoogLeNet.

Meanwhile, in terms of time efficiency, the VMD-GoogLeNet method spends the least time because it does not use intelligent algorithms for parameter optimization. Compared with PSO and WOA, the IWHO method has moderate time cost, but the highest accuracy.

**Figure 12:**
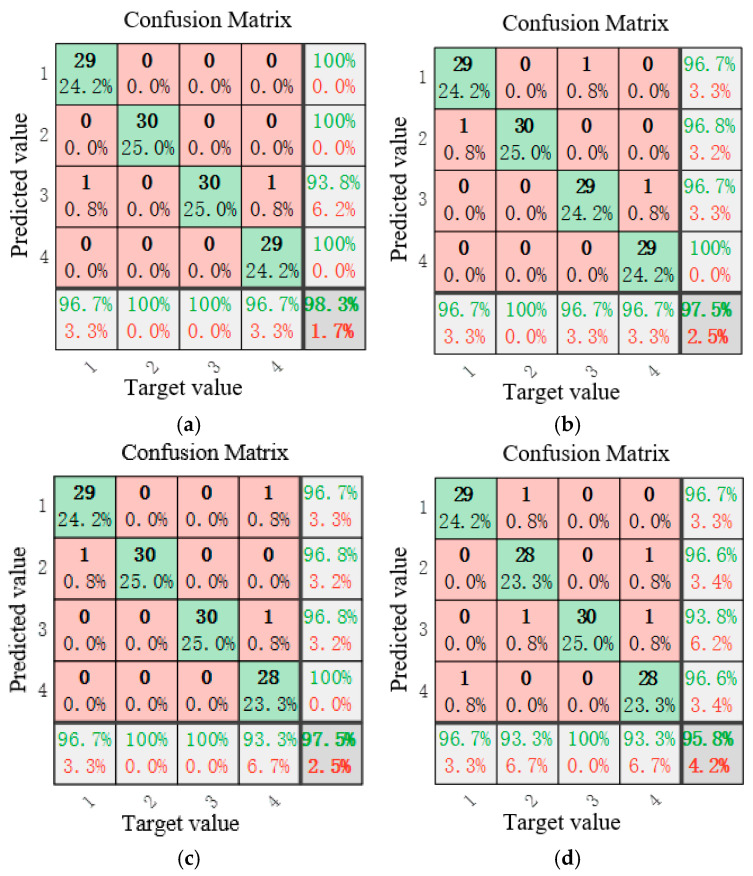

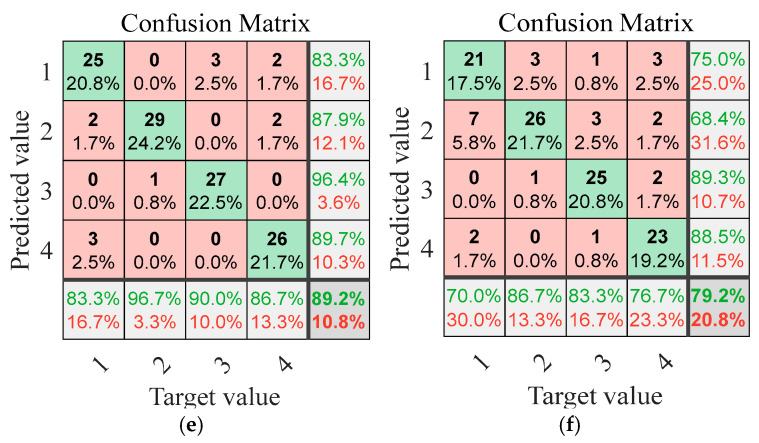
Confusion matrices of fault diagnosis results under different SNRs for case study I (green: correct diagnosis, red: incorrect diagnosis). (**a**) SNR = 20. (**b**) SNR = 15. (**c**) SNR = 10. (**d**) SNR = 5. (**e**) SNR = 0. (**f**) SNR = −5.

To evaluate the model’s robustness under realistic industrial conditions where measurement noise is inevitable, we systematically introduced Gaussian white noise at different signal-to-noise ratios (SNRs: −5 dB, 0 dB, 5 dB, 10 dB, 15 dB, and 20 dB) to the experimental vibration signals across all four operational states. The diagnostic performance of the IWHO-VMD-GoogLeNet method under these noisy conditions is comprehensively presented in Figure 12, providing critical insights into its practical applicability in real-world environments with varying noise levels. For clarity, correct and incorrect classifications in the fault diagnosis results are highlighted in green and red, respectively, in Figure 12, and in all subsequent figures that present fault-diagnosis results. This rigorous testing protocol validates not only the method’s superior accuracy but also its operational reliability when handling contaminated vibration data.

Figure 12 reveals two key observations regarding the method’s performance under noise interference: (1) The diagnostic accuracy exhibits a gradual decline with decreasing signal-to-noise ratio (SNR). Specifically, the accuracy reaches 98.30% at SNR = 20 dB, while decreasing to 95.80% at SNR = 5 dB, demonstrating the expected inverse relationship between noise intensity and classification performance. (2) When the SNR is 0 dB or negative, the diagnostic accuracy shows a significant decline, as the noise energy greatly exceeds the signal energy. However, even in this case, when the SNR is −5 dB, the diagnostic accuracy can still reach 79.2%. (3) Despite these challenging noise conditions, the proposed IWHO-VMD-GoogLeNet method maintains robust diagnostic capability, consistently achieving high accuracy levels across the tested SNR range. These results confirm the method’s strong noise immunity and reliability in practical applications where signal quality may be compromised.

## 4. Case Study II: HUST Dataset

To further verify the effectiveness of the proposed method, a fault diagnosis study of rolling bearings was conducted using a practical dataset from Hanoi University of Science and Technology (HUST).

### 4.1. Data Acquisition for Case Study II

**Figure 13:**
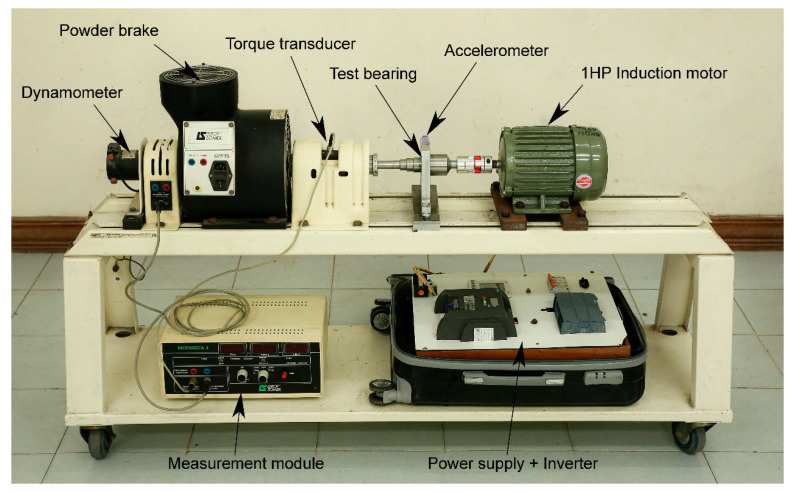
HUST testing rig.

The basic layout of the HUST test bench is shown in Figure 13.

**Figure 14:**
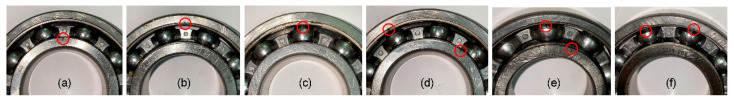
Fault types of rolling bearings (red circle denotes fault location). (**a**) Inner race fault. (**b**) Outer race fault. (**c**) Ball fault. (**d**) Inner and outer race faults. (**e**) Inner race and ball faults. (**f**) Outer race and ball faults.

As shown in Figure 13, the test bench consists of a 750 W (1 HP) induction motor driving a multi-stage shaft, and a powder brake manufactured by Leroy Somer, which serves as a simulated load. The motor is controlled by a variable frequency drive, and the powder brake acts as a controllable resistance. In addition, a torque sensor and a dynamometer are installed on the shaft to monitor the motor’s load and speed. Faulty bearings are mounted on various types of bearing housings that can be flexibly replaced along the multi-stage shaft. A PCB 325C33 accelerometer (Depew, NY, USA) is mounted vertically on the bearing to measure vibration. The bearing defects in the dataset were artificially induced. The bearings used to generate defects include the ID 6204, 6205, 6206, 6207, and 6208 ball bearings from KG Bearing India (New Delhi, India). The dataset includes common single faults such as inner race fault, outer race fault, and ball fault. In addition, compound faults include combinations such as inner and outer race faults, inner race and ball faults, and outer race and ball faults (as shown in Figure 14).

The data acquisition system consists of an accelerometer, a measurement module, a chassis, and LabVIEW software 2021. The dataset includes vibration data from 27 prototype defective bearings and 3 healthy bearings under three different load conditions: 0 W, 200 W, and 400 W. All data were collected at a high sampling rate of 51.2 kHz. The proposed method was validated using datasets corresponding to normal bearings, inner race faults, outer race faults, and rolling element faults.

### 4.2. Fault Diagnosis Results and Performance Comparison of Methods

**Figure 15:**
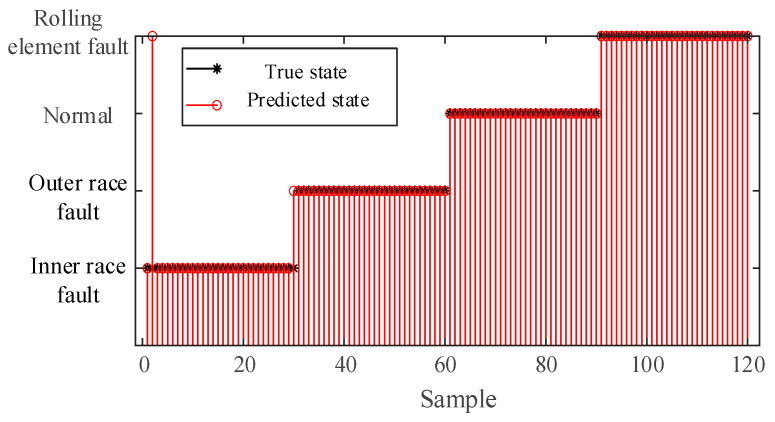
Fault diagnosis results of IWHO-VMD-GoogLeNet for case study II.

**Figure 16:**
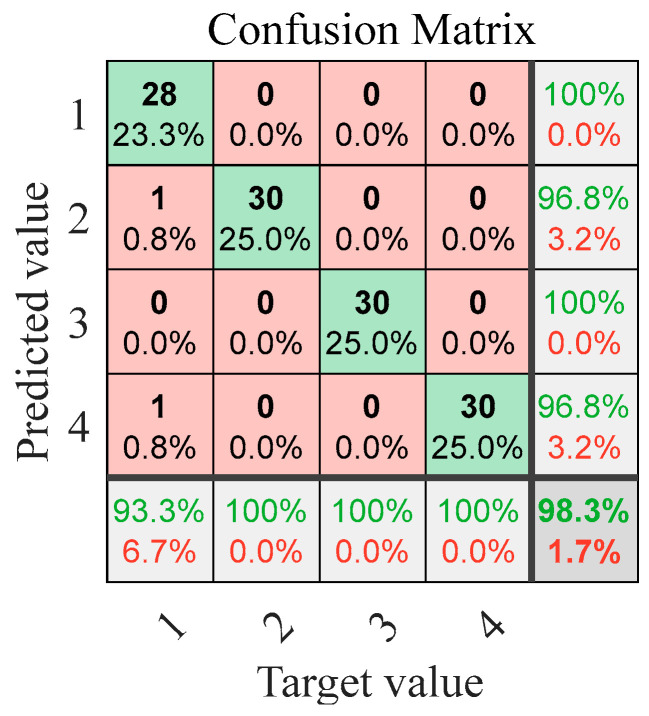
Confusion matrices of fault diagnosis results of IWHO-VMD-GoogLeNet for case study II (green: correct diagnosis, red: incorrect diagnosis).

The constructed feature vectors were input into the GoogLeNet model for training to achieve fault diagnosis of rolling bearings. The fault diagnosis results and their corresponding confusion matrix are presented in Figure 15 and Figure 16.

It can be seen from Figure 15 and Figure 16 that all test samples corresponding to the normal condition, outer race fault, and rolling element fault were correctly classified, achieving perfect diagnostic accuracy (100%). For the inner race fault condition, 28 out of 30 test samples were correctly identified, with one sample misclassified as an outer race fault and another as a rolling element fault, resulting in a diagnostic accuracy of 93.33%. Overall, the system achieved a diagnostic accuracy of 98.33%, with only 2 misclassifications out of a total of 120 test samples across the four operating conditions.

**Figure 17:**
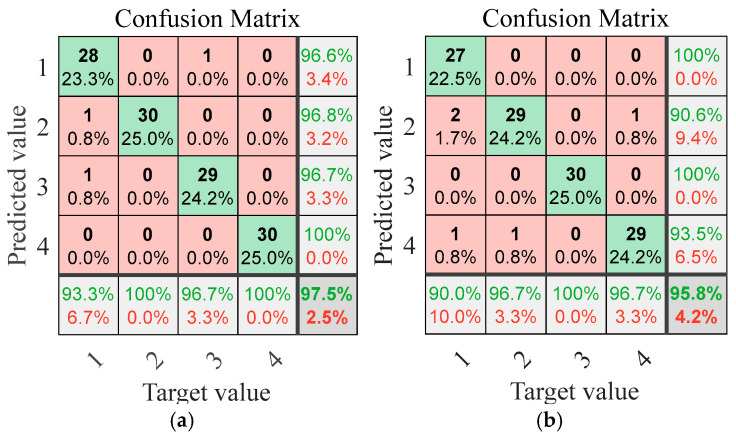

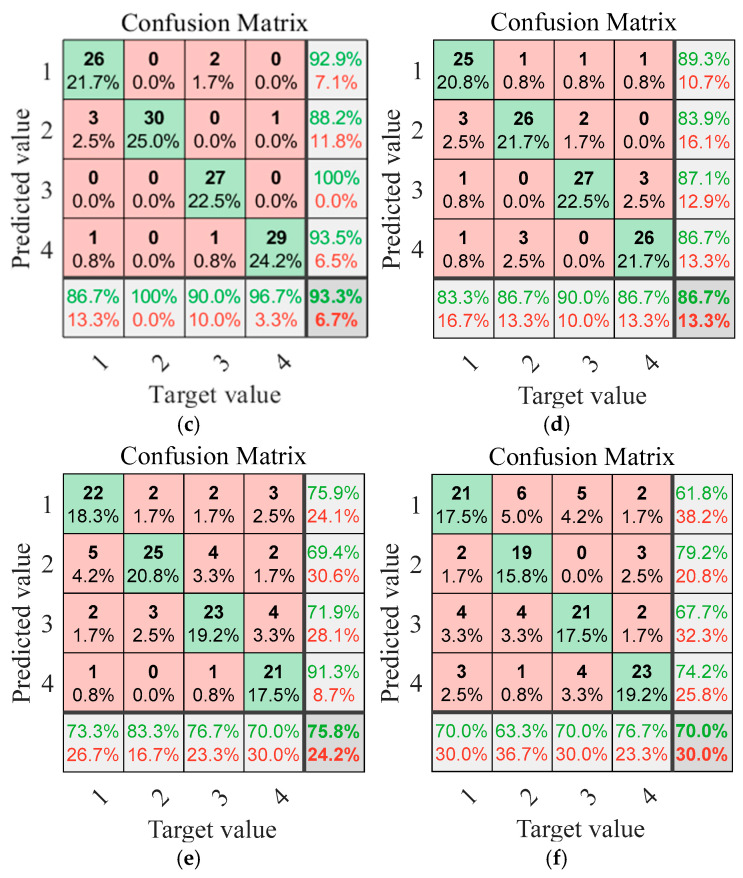
Confusion matrices of fault diagnosis results under different SNRs for case study II (green: correct diagnosis, red: incorrect diagnosis). (**a**) SNR = 20. (**b**) SNR = 15. (**c**) SNR = 10. (**d**) SNR = 5. (**e**) SNR = 0. (**f**) SNR = −5.

Similarly to Case 1, to verify the model’s robustness in actual noisy environments, we designed experiments under different SNRs. The SNR settings are the same as those in Case 1. The diagnostic performance of the IWHO-VMD-GoogLeNet method under these noisy conditions is comprehensively presented in Figure 17.

From Figure 17, similar conclusions to those of Figure 12 can be drawn: the diagnostic accuracy shows a gradient decline with the decrease in signal-to-noise ratio (SNR). Specifically, the accuracy reaches 97.50% at SNR = 20 dB, and decreases to 86.70% at SNR = 5 dB, which confirms that the negative correlation between noise intensity and classification performance is in line with theoretical expectations. It is worth noting that even in the extreme noisy environment with SNR = −5 dB, the diagnostic accuracy still remains at 70%. The above experimental data fully demonstrate that the method has significant anti-noise robustness and reliability in practical engineering applications, especially in scenarios where signal quality may deteriorate.

## 5. Conclusions

To address the challenges in condition monitoring and fault diagnosis of mechanical equipment, this study proposes a novel fault diagnosis method that combines improved VMD with GoogLeNet, namely the IWHO-VMD-GoogLeNet approach. The research demonstrates the following: (1) The integration of optimized VMD parameters through the IWHO algorithm with GoogLeNet classifier significantly enhances the accuracy of bearing fault pattern recognition. (2) The proposed IWHO algorithm enables adaptive optimization of critical VMD parameters (mode number *K* and penalty factor *α*), overcoming the limitations of empirical parameter setting in conventional VMD and effectively mitigating mode mixing issues. Most notably, the IWHO-VMD-GoogLeNet method achieves the highest fault diagnosis accuracy of 99.17%, representing a 5.31% improvement over VMD-GoogLeNet, a 3.49% enhancement compared to PSO-VMD-GoogLeNet, and a 2.59% increase versus WOA-VMD-GoogLeNet. (3) Noise interference tests with varying signal-to-noise ratios (SNRs) confirm the adaptability of the IWHO-VMD-GoogLeNet method in simulated industrial noise environments. The feature extraction strategy based on improved VMD and kurtosis criterion demonstrates effective noise suppression while maintaining feature discriminability and high diagnostic accuracy.

Although the proposed method exhibits superior diagnostic accuracy and robustness in case studies, several aspects warrant further improvement: (1) While the kurtosis criterion effectively captures impact characteristics, it may overlook other potential fault signatures. (2) The model’s generalization capability for coupled faults or unknown fault types requires additional investigation. (3) Future research should explore adaptive feature selection strategies incorporating multi-domain indicators to enhance the model’s generalization performance. (4) Validating the method on larger and more diverse test datasets and samples will further strengthen confidence in its robustness and effectiveness.

## Figures and Tables

**Table 1:** The time-domain, frequency-domain, and time–frequency-domain features.

Domain	Features
Time-domain	Mean, variance, standard deviation, kurtosis, skewness
Frequency-domain	Center frequency, mean frequency, power spectrum
Time–frequency-domain	Energy entropy

**Table 2:** Optimized VMD parameters obtained by IWHO under four conditions.

Condition	Rolling Element Fault	Outer Race Fault	Inner Race Fault	Normal State
Penalty Factor (α)	3000	1000	1000	1609
Mode Number (K)	10	10	7	7

**Table 3:** Comparative analysis of fault diagnosis accuracy across different methods.

Model	VMD-GoogLeNet	PSO-VMD-GoogLeNet	WOA-VMD-GoogLeNet	IWHO-VMD-GoogLeNet
Accuracy (%)	94.17	95.83	96.67	99.17
Time(s)	104.00	322.29	863.24	558.36

## Data Availability

Data are available on request from the authors.
